# Advance Care Planning in Glioblastoma Patients

**DOI:** 10.3390/cancers8110102

**Published:** 2016-11-08

**Authors:** Lara Fritz, Linda Dirven, Jaap C. Reijneveld, Johan A. F. Koekkoek, Anne M. Stiggelbout, H. Roeline W. Pasman, Martin J. B. Taphoorn

**Affiliations:** 1 Department of Neurology, Haaglanden Medical Center, P.O. BOX 432, 2501 CK The Hague, The Netherlands; FriLar@mchaaglanden.nl (L.F.); j.a.f.koekkoek@lumc.nl (J.A.F.K.); 2 Department of Neurology, Leiden University Medical Center, P.O. BOX 9600, 2300 RC Leiden, The Netherlands; l.dirven@lumc.nl; 3 Department of Neurology, VU University Medical Center, P.O. BOX 7057, 1007 MB Amsterdam, The Netherlands; jc.reijneveld@vumc.nl; 4 Department of Neurology, Academic Medical Center, P.O. BOX 22660, 1100 DD Amsterdam, The Netherlands; 5 Department of Medical Decision Making/Quality of Care, Leiden University Medical Center, P.O. BOX 9600, 2300 RC Leiden, The Netherlands; a.m.stiggelbout@lumc.nl; 6 Department of Public and Occupational Health, VU University Medical Center, P.O. BOX 7057, 1007 MB Amsterdam, The Netherlands; hrw.pasman@vumc.nl

**Keywords:** glioblastoma, advance care planning, decision-making, end-of-life, palliative care

## Abstract

Despite multimodal treatment with surgery, radiotherapy and chemotherapy, glioblastoma is an incurable disease with a poor prognosis. During the disease course, glioblastoma patients may experience progressive neurological deficits, symptoms of increased intracranial pressure such as drowsiness and headache, incontinence, seizures and progressive cognitive dysfunction. These patients not only have cancer, but also a progressive brain disease. This may seriously interfere with their ability to make their own decisions regarding treatment. It is therefore warranted to involve glioblastoma patients early in the disease trajectory in treatment decision-making on their future care, including the end of life (EOL) care, which can be achieved with *Advance Care Planning* (ACP). Although ACP, by definition, aims at timely involvement of patients and proxies in decision-making on future care, the optimal moment to initiate ACP discussions in the disease trajectory of glioblastoma patients remains controversial. Moreover, the disease-specific content of these ACP discussions needs to be established. In this article, we will first describe the history of patient participation in treatment decision-making, including the shift towards ACP. Secondly, we will describe the possible role of ACP for glioblastoma patients, with the specific aim of treatment of disease-specific symptoms such as somnolence and dysphagia, epileptic seizures, headache, and personality changes, agitation and delirium in the EOL phase, and the importance of timing of ACP discussions in this patient population.

## 1. Introduction

Glioblastomas are the most malignant and most commonly occurring gliomas, with an annual incidence of approximately 3 per 100,000 persons [1,2]. Gliomas are primary brain tumors originating from glial cells of the central nervous system, which rarely disseminate outside the nervous system but grow diffusely into the surrounding brain tissue. This characteristic growth pattern attributes to the nearly always incurable nature of gliomas. Previously, the classification of these tumors was mainly based on histological features (World Health Organisation (WHO) 2007 criteria) [3] resulting in low-grade and high-grade gliomas, with a higher grade being more malignant. This is reflected in the median survival of patients: low-grade glioma patients have a median survival of 6 to 13 years [4], while patients with glioblastoma have a median survival of only 15 months [5]. Recently, the WHO classification has been substantially revised, now including molecular parameters in addition to histology to define tumor entities [6]. These new criteria allow better prediction of response to treatment and prognosis. Other prognostic factors for survival are age, performance status, tumor location, and extent of surgical resection [7]. Histology features, molecular parameters, and other prognostic factors may affect the choice of treatment.

Glioblastoma is still incurable, despite multimodal treatment with surgery, radiotherapy and chemotherapy. Patients may present with various symptoms and signs. Increased intracranial pressure, resulting from a rapidly growing tumor, can manifest itself in progressive headache, nausea, vomiting, drowsiness, and visual abnormalities. Other symptoms are partial or generalized seizures, progressive focal neurologic deficits and cognitive decline [8]. Glioblastoma patients not only have cancer, but also a progressive brain disease, and are therefore different from patients with other cancers or patients with cognitive impairment or dementia. The fact that glioblastoma patients have a progressive brain disease may seriously interfere with their ability to make their own decisions regarding treatment. Soon after diagnosis, about half of the glioma patients already have problems in making treatment decisions [9], and this percentage increases when death approaches [10]. It is therefore warranted to involve glioblastoma patients early in the disease trajectory in treatment decision-making, which can be achieved with Advance Care Planning (ACP).

In this article, we will first describe the history of patient participation in treatment decision-making, including the shift towards ACP. Secondly, we will describe the possible role of ACP for glioblastoma patients, with the specific aim of symptom treatment in the End Of Life (EOL) phase, and the importance of timely ACP discussions in this patient population.

## 2. Patient Participation in Decision-Making and ACP

During recent decades, patient autonomy has become increasingly important and many patients wish to be involved in treatment decision-making [11]. Traditionally, physicians were expected to make the best clinical decisions and patients were marginally involved in the decision-making process. Over time, a more patient-centered approach evolved in medicine, leading to an enhanced involvement of patients in clinical decision-making [10,12,13,14].

Several studies have shown discrepancies between preferred roles in clinical decision-making on the one hand, and actual or perceived roles on the other hand [11,15]. A systematic review involving patients with various types of cancers demonstrated that patients’ involvement in decision-making occurred to a lesser degree than they preferred [11]. Specifically, patients with breast, prostate, colorectal, lung and gynecologic cancers wanted a more shared or an active role in decision-making [11].

Initially, patients were stimulated to actively participate in treatment decision-making by completing advance directives (ADs), which was stimulated by the implementation of The Patient Self-Determination Act (PSDA) in 1991 [16]. However, ADs as such do not empower patients to make decisions in (complex) medical situations [17,18]. Although it is important to identify patient’s expectations, wishes and preferences, these need to be adequately communicated to proxies [17] (e.g., partners or relatives) as well as physicians in order to be effective in the decision-making process. Evidence for the effectiveness of ADs in daily clinical practice is therefore still ambiguous. Medical situations, particularly at the EOL, might be too unpredictable or too complex for ADs [17,18]. In addition, proxies may not be aware of patients’ preferences or do not want to pursue these wishes because they are not comfortable with their role or their lack of knowledge [18]. However, if communicated appropriately to all persons involved, ADs may be effective: it has been shown that the actually supplied care at the EOL is strongly associated with the patients’ preferences as specified in the AD [19].

Most studies on patients’ preferences for participation in clinical decision-making are conducted in the curative setting, limiting evidence for the palliative care setting, which includes the EOL phase. Nevertheless, Brom et al. [20] found that 93% of the patients prefer to share responsibility with their physician in clinical decision-making in the palliative setting. The other 7% preferred to be informed about the treatment options, but choose to leave the decision to their physician. In addition, individual patients’ preferences may change during the disease trajectory, along with the preferred level of participation in decision-making [20]. When the treatment aim is to prolong life, patients would tend to leave the decision to their physician because of their lack of expertise. However, a shift towards a more active participation in decision-making will occur when maintaining quality of life would become the most important treatment goal [20]. For this reason, it is important to communicate openly about patient’s expectations, wishes, and preferences for participation during the entire disease trajectory [20].

More recently, there has been a shift towards a broader process of communication than the completion of an AD alone, referred to as *Advance Care Planning* (ACP). ACP is a process aimed at timely involvement of patients and their relatives in decision-making on future (palliative) care, including EOL care [21]. Although ACP aims at timely involvement, it is often not initiated early enough in daily clinical practice, because of barriers in physicians and patients’ behavior, and the organizational (e.g., ACP documentation not being available at the appropriate time) and legal context [22]. For example, physicians have the tendency to avoid this subject, as they are afraid that discussing death and dying will take away hope [23]. However, timely initiation of ACP is important, because it gives patients time to evaluate all care options and communicate their preferences. It allows patients, their proxies and physicians to proactively address the challenges together during the course of disease. Two studies, the first one including elderly patients and the second one including patients with congestive heart failure or end-stage renal disease, have shown that patients who participated in ACP discussions appreciated having such discussions [24,25]. However, other studies have reported that not all patients prefer to participate in ACP discussions [21,26,27]. Barnes et al. showed that although most palliative care and oncology participants appreciated ACP discussions, some were not ready for it yet [26]. Likewise, Andreassen et al. found that patients experienced participating in ACP discussions differently; some patients felt more relieved, more confident and in control, while other patients found ACP not relevant or were of the opinion that their wishes would not be met [21].

For cancer patients in their EOL phase, there is an increasing body of evidence that early palliative care is effective in improving health related quality of life and mood [28,29]. Improved health related quality of life and mood may also be achieved through ACP. Models of ACP have shown that a coordinated, systematic, patient-centered approach to ACP by trained non-medical facilitators can improve outcomes for patients [24,30,31]. A randomized clinical trial in elderly patients showed that facilitated ACP improves both the quality of EOL care, as well as patient and family satisfaction, and also reduces stress, anxiety, and depression in surviving relatives [24]. A study in patients with diseases such as congestive heart failure, end-stage renal disease, and chronic obstructive pulmonary disease has shown that facilitated ACP increased the level of agreement between the patients’ preference for care and the actually received care [25].

Although timely initiation of ACP is warranted, it is unclear what the optimal timing is [22,32,33]. The timing to discuss ACP has been found to influence its acceptability and effect [22,26]. In a study including terminally ill patients it was found that the best timing of introducing early palliative care was soon after diagnosis [29]. However, in a small study that included cancer patients in all disease stages, the majority of patients felt that the best timing of introducing ACP was after disease recurrence, failure of curative treatment, or at the time it became evident that the prognosis was poor [26]. In contrast to the previous study, this study showed that it was inappropriate to initiate ACP around the time of diagnosis or during active treatment [26]. Nevertheless, for patients with an incurable disease, timely initiation of ACP is particularly warranted. In addition, these patients may experience a rapid decline in their clinical condition, obviating decision-making. On the other hand, for patients who are treated with curative intent and who have a chance to be cured, it is particularly unclear whether early initiation of ACP is desirable. In the latter case, patients may become distressed or confused discussing (EOL) care issues early on.

## 3. Relevance of ACP in Glioblastoma Patients

Although little is known about the effect of ACP in glioblastoma patients, Walbert et al. [34] suggested that early palliative care planning through structured ACP could improve symptom control and quality of life in glioblastoma patients. This suggests that it is important to discuss the occurrence and treatment of disease-specific symptoms in an ACP program for glioblastoma patients. Moreover, it was found that if glioma patients expressed their preferences on EOL care, these were often met (90%) [35]. Furthermore, the majority of glioma patients died in their preferred place of death if this was communicated [36]. Dying in the preferred place of death was also associated with dignity [37]. Although glioblastoma patients not only experience progressive cognitive impairments, but also have cancer, it was found that ACP reduced inappropriate hospital admissions and health-care costs in patients with cognitive impairment or dementia [38]. Thus, facilitated ACP could potentially contribute to a better quality of care and quality of life in glioblastoma patients.

As mentioned, early implementation of ACP may be particularly warranted for glioblastoma patients, because these patients have an incurable disease and most of them will at some point no longer be able to participate in ACP discussions due to the rapid cognitive decline that may occur as the disease progresses. Indeed, Kerrigan et al. found that the majority (64%) of glioblastoma patients were mentally incapacitated prior to surgery [39], hampering decision-making. This underlines that early implementation of ACP discussions is warranted in this unique patient population. Similarly, early implementation of ACP is also warranted in patients with cognitive impairment or dementia [38]. On the other hand, a previous study [26] has shown that the best timing to initiate ACP discussions was at recurrence or when curative treatment failed, suggesting that ACP should not be introduced around diagnosis. What the optimal timing of initiating ACP discussions is for glioblastoma patients therefore requires further research. Experts in the field of palliative care for glioblastoma have suggested that ACP could for example be introduced at the end of chemoradiation (12–16 weeks after the diagnosis), after the first three courses of the adjuvant chemotherapy (about six months after the diagnosis) or after the adjuvant chemotherapy (about nine months after diagnosis).

Irrespective of the optimal timing of ACP in the care of glioblastoma patients, the content of the ACP discussion is also important. Below we will discuss topics that may be relevant for glioblastoma patients, and because of the incurable nature of the disease we will focus on the EOL phase. In the next section, we will first discuss the possible role of ACP in relation to the treatment of the main disease-specific symptoms of glioblastoma patients in the EOL phase, including a description of current best practices or recommendations. Next, we will discuss other important topics related to this phase that are relevant for ACP, which will be more general and applicable to all type of patients.

## 4. Possible Role of ACP in the Treatment of the Main Symptoms in the EOL Phase

Even though no clear definition exists, the EOL phase in glioma patients is often confined to the last three months of life [40,41]. Treatment goals in the EOL phase are to prolong survival, maintain a satisfactory quality of life, and to ensure prevention and relief of suffering [42].

Primary brain tumor patients may experience severe symptoms due to the progressive disease as well as to adverse effects of treatment, requiring adequate palliative care [43,44,45,46]. Disease-specific symptoms in the last weeks of the EOL phase include epilepsy, headache, drowsiness, dysphagia, and cognitive disturbances like agitation and delirium [41,45,46,47]. These symptoms occur more often in glioma patients than general EOL symptoms as commonly seen in patients with systemic cancers, such as fatigue, pain, nausea, constipation and dyspnea [41,47,48]. Glioma patients with more symptoms in the week preceding death experience a lower quality of care compared to patients with fewer symptoms [48]. In addition, motor disability and cognitive decline negatively contribute to the patient’s quality of life and social well-being. Confusion and seizures are symptoms that prevent patients from dying peacefully [45]. A lack of control of symptoms may also lead to more transitions to different health care settings, which may subsequently prevent patients from dying with dignity [37].

The high symptom burden in glioblastoma patients necessitates appropriate palliative care in the EOL phase [46]. ACP could facilitate timely and adequate amelioration of symptoms in the EOL phase by discussing possible options in different medical situations. This may in turn result in higher satisfaction with care, better quality of life, and lower levels of anxiety and depression in both patients and their caregivers. Adequate care also may help to decrease caregiver burden, which was found to be high in the EOL phase [41].

### 4.1. Somnolence and Dysphagia

When the EOL approaches, an increasing number of glioma patients will become somnolent or even lose consciousness [45,48]. Dysphagia as a consequence of somnolence and loss of consciousness, may particularly hamper the regular intake of oral drugs [45,48]. Three months before death, somnolence and dysphagia were present in 31.2% and 7.5% of high-grade glioma patients, respectively, and these percentages are more than doubled one week before death (74.8% and 24.5%, respectively). Moreover, 81.2% of glioma patients in whom medication was withdrawn, were somnolent and/or had dysphagia [48]. Apart from the medication, the oral intake of food and fluids will also be impaired in these patients [45].

Early discussion of this topic with patients and their proxies may help to inform them about the consequences of withholding or withdrawing drugs, nutrition and/or fluids. In addition, the patients’ wishes with respect to withdrawing or withholding artificial administration of nutrition and fluids may be discussed and recorded in an AD.

### 4.2. Epileptic Seizures

Seizures are common in the EOL phase, with approximately 30% of glioma patients suffering a seizure during the last week before death, both in patients with and without a history of seizures [47,49]. Moreover, half of the patients taper anti-epileptic drugs (AEDs) in the last week of life, of whom approximately one third experiences seizures in that week [49]. Indeed, optimal anticonvulsive treatment seems to be difficult towards death in many brain tumor patients, because of swallowing difficulties or somnolence [50]. This may lead to a higher risk of seizures in the last days before death. As a consequence, patients with new seizures may be admitted to the hospital for treatment, resulting in distress for both patients and caregivers. Administration of AEDs therefore needs to be continued during the entire EOL phase, particularly in patients with a higher risk of seizures.

One option to continue treatment is that oral AEDs are substituted through other routes, such as nasal, buccal, subcutaneous or intravenous routes, as soon as swallowing difficulties develop. Intranasal and intrabuccal applications are most convenient since these can be administered by the caregiver in the out-of-hospital setting [51,52]. While intranasal midazolam and rectal diazepam show similar efficacy in the treatment of acute seizures, caregivers of patients with glioma are clearly more satisfied with the use of intranasal midazolam [51].

Treatment of seizures in the EOL phase is a topic that is eminently suitable to discuss in the context of ACP. Information can be provided on the occurrence and risks of seizures in the EOL phase, which may reduce distress in both patients and caregivers. In addition, alternative methods of administrating AEDs can be discussed early in the disease trajectory. This may subsequently lead to better seizure control in the EOL phase, and prevention of hospitalization.

### 4.3. Headache

Headache is a frequently reported symptom in the EOL phase of glioma patients [41,45,46,48]. In the majority of patients, headache is mild and intermittent [45]. Headache is often caused by increased intracranial pressure due to the tumor (recurrent growth) itself, vasogenic edema surrounding the brain tumor, and sometimes by a concomitant hydrocephalus. Headache can be alleviated with pain medication with (non-)opioids or treatment with corticosteroids [45] (typically dexamethasone), in order to reduce vasogenic edema. Conversely, due to adverse effects of dexamethasone such as gastro-intestinal problems, psychiatric problems and/or proximal muscle atrophy and weakness, the lowest effective dose should be prescribed in order to retain the highest quality of life [53].

As with other oral drugs, administration of dexamethasone is often discontinued in the EOL phase due to progressively decreased consciousness. However, acute withdrawal of dexamethasone may result in recurrence of symptoms, a further decrease of consciousness and acute adrenal insufficiency [53]. Withdrawal of dexamethasone in unconsciousness patients may also be intended to hasten death when life is no longer considered meaningful [10]. Withdrawal of dexamethasone is a suitable topic to discuss in the context of ACP. Information on the benefits and risks can be provided, which may reduce stress in both patients and caregivers.

### 4.4. Personality Changes, Agitation and Delirium

Prevalence of changes in behavior and personality in glioma patients ranges between 8% and 67% [54] and was found to be associated with distress and a lower quality of life as perceived by both patients and relatives [55,56]. Spouses are often more distressed by the change in the patient’s personality than patients themselves [57]. Behavioral changes or delirium occurring shortly before death may hamper a “peaceful” process of dying and the patient’s agitation or delirium in the last hours of life can be very distressing for family members [45].

Several options are considered appropriate in the treatment of these symptoms, such as opioids, sedatives and antipsychotics and particularly opioids and sedatives, which are commonly described in the EOL phase of glioma patients [48]. ACP could help to inform patients and proxies about the advantages and disadvantages of these treatment options.

### 4.5. Other Issues for ACP Discussions in Glioblastoma

Other topics that may be discussed in ACP sessions are the health condition of the patient, worries of the patient, the possibilities and impossibilities of (palliative) treatment, and the preferred place of care and death. These topics are not specific for glioblastoma patients, but applicable to all type of patients.

Palliative sedation, physician-assisted suicide or euthanasia might also be discussed during ACP sessions. Palliative sedation refers to the administration of sedative drugs without the intention to shorten life and is often used to treat delirium and agitation in terminally ill patients [58]. Palliative sedation is different from physician-assisted suicide (PAS) or euthanasia, in which drugs are administered with the intention to hasten death [59]. In some countries euthanasia and PAS are legal and could be specified in an AD. Patient preferences on palliative sedation could also as such be specified in an AD.

## 5. Conclusions

In conclusion, timely initiation of ACP is eminently important for glioblastoma patients, because these patients not only have an incurable disease, but also experience progressive cognitive impairments over time, interfering with their ability to make clinical decisions. Currently, little is known about the effect of ACP in glioblastoma patients. Therefore, more research is needed to further establish which topics are important to discuss during the ACP sessions, what the optimal timing for initiation of an ACP program is, and whether implementation of an ACP program is feasible and effective in this patient population.
